# Evaluation of Drug and Alcohol Abuse in People Aged 15 Years and Older in Iran

**DOI:** 10.18502/ijph.v49i10.4697

**Published:** 2020-10

**Authors:** Ahmad Ali NOORBALA, Armita SALJOUGHIAN, Seyed Abbas BAGHERI YAZDI, Elham FAGHIHZADEH, Mohammad Hadi FARAHZADI, Koorosh KAMALI, Soghrat FAGHIHZADEH, Ahmad HAJEBI, Shahin AKHONDZADEH, Mir Taher MOUSAVI

**Affiliations:** 1. Department of Psychiatry, Psychosomatic Medicine Research Center, Imam Khomeini Hospital Complex, Tehran University of Medical Sciences, Tehran, Iran; 2. Psychosomatic Medicine Research Center, Imam Khomeini Hospital Complex, Tehran University of Medical Sciences, Tehran, Iran; 3. Department of Mental Health, Ministry of Health and Medical Education of Iran, Tehran, Iran; 4. Department of Biostatistics, Paramedical School, Shahid Beheshti University of Medical Sciences, Tehran, Iran; 5. Research Center of Addiction and Behavioral Sciences, Shahid Sadoughi University of Medical Sciences, Yazd, Iran; 6. Department of Public Health, School of Public Health, Zanjan University of Medical Sciences, Zanjan, Iran; 7. Department of Biostatistics and Epidemiology, School of Medicine, Zanjan University of Medical Sciences, Zanjan, Iran; 8. Research Center for Addiction and Risky Behaviors (ReCARB), Psychiatric Department, Iran University of Medical Sciences, Tehran, Iran; 9. Psychiatric Research Center, Roozbeh Hospital, Tehran University of Medical Sciences, Tehran, Iran; 10. Department of Sociology, University of Welfare, Tehran, Iran

**Keywords:** Drug and alcohol abuse, Smoking, Iran

## Abstract

**Background:**

Drug addiction is known as one of the health, medical and social problems of the present century. Beyond the harmful physical and mental consequences for addicts, drug abuse can cause serious social problems. The aim of this study was to evaluate drug and alcohol abuse in individuals aged 15 yr and over in Iran in 2015.

**Methods:**

This descriptive cross-sectional study was done on individuals aged 15 yr and older who lived in urban and rural areas of Iran. Overall, 36,600 individuals were selected by systematic and cluster random sampling. The postal code was used to access the samples in 31 provinces of Iran. In each province 1200 individuals (50% men, 50% women) were evaluated. The used instrument was the Alcohol, Smoking and Substance Involvement Screening Test (ASSIST), and data were analyzed using SPSS software.

**Results:**

The results showed 4.6% of individuals used Opium and its derivatives, 0.4% used Cannabis, 0.4% used Amphetamine stimulants, 6.1% used Sedative drugs, and 2.6% used Alcoholic beverages on a regular basis. Within the last 12 months, 3.9% of individuals used Opium and its derivatives, 0.4% used Cannabis, 0.3% used Amphetamine stimulants, 5.8% used Sedative drugs, and 1.9% used Alcoholic beverages. With the exception of Sedatives drugs men used more drugs than women and residents of rural areas used more opium and its derivatives than other groups of drugs. Based on the provincial distribution, Kerman and Qom used the highest and lowest prevalence percentage of Opium and its derivatives respectively.

**Conclusion:**

Overall, 2340000 individuals were addicts used Opium and its derivatives in 2015, therefore, medical and health officials should take all necessary measures to deal with these serious social problems.

## Introduction

Drug addiction is recognized as one of the main health, medical and social problems of this century, which can lead to severe physical, psychological and social damages (1, 2). The devastating impact of these substances on the health of individuals has caused a lot of social and economic costs, increasing of crime and death in the world, thus it has become a major threat to societies (3, 4). The common boundaries between Iran and the two main drug producing countries, Afghanistan and Pakistan, has made Iran the easiest platform to transport drugs to European countries. In this process some of these drugs are distributed by the profitable people and caused addiction in Iran (5).

Iran has been dealing with addiction and drug distribution and has tried to prohibit domestic producers by punishing drug importers and distributors (6). According to the World Drug Report (2017), prevalence of drug use in the world is increasing. In 2006, 4.9%, in the year 2014, 5.2% and in 2015, 5.3% of the population aged 15–64 yrs. had at least one substance use (7). Review of researches performed in the last four decades in Iran, show an increasing trend in the use and abuse of drug (8, 9). However, this process has fluctuated due to the difference in the statistical population, the use of various tools and methods of implementation. In the first study of the rapid assessment of substances in Iran, the rate of addicts in the country is estimated at about 700,000 people (10). In an epidemiological study of drug abuse in Iran, estimated the number of opium and its derivatives about 3,700,000 people, which 2,547,000 of them have had addiction (11). In the second study of rapid assessment of substance abuse and drug dependence in Iran, the number of addicts in the country ranged from 1,200,000 to 1,800,000 (2.4 to 2.65%) (12). In the third national study of rapid assessment of substance abuse and drug dependence in Iran, estimated the number of opium and other opiate users about 1,100,000 to 1,200,000 people (13). In the National Survey of Mental Health, estimated that the prevalence of any drug and alcohol-related disorders over the past 12 months, in the general population of 15–64 yrs., was 2.8% (14). The results of the National Survey on drug users’ prevalence among Iranian citizens showed that the prevalence of drug users was 2.65% and the number of consumers was 1,355,000 people (15). The increase of AIDS among injecting drug users as well as increasing social and economic problems of addiction in the community has led Iran to take fundamental steps to prevent the drug use and provide appropriate treatment and rehabilitation for volunteers to be under addiction treatment (16).

This research was carried out as part of the National Survey of Mental Health and Social Capital in 2015 (17) and the aim of this study was to determine the status of drug and alcohol abuse in Iran.

## Materials and Methods

This descriptive cross-sectional study was done within the framework of Mental Health and Social Capital survey in Dec and Jan 2015. The sample included individuals, aged 15 yrs. and older living in urban and rural areas of Iran. The systematic and cluster randomized sampling was used to do the study. The sample size was estimated by formula of sample size calculation in cross-sectional studies to determine the status of the society mental health and included 1200 individuals in each province. The postal code was used to access the samples in rural and urban provinces (the province center with two cities) of Iran.

In addition to demographic questions, Alcohol, Smoking and Substance Involvement Screening Test (ASSIST) questionnaire was used. This questionnaire was designed by a group of WHO experts in 2003. It includes 8 items to screen drug abuse in primary health care settings (18–20).

The questionnaire was filled out by mental health experts of the health departments of each province. The consent form was completed to consider ethical principles. Moreover, participants name and surname were not recorded in the questionnaire. The questioner team including a man and a woman referred to the house according to the postal code and evaluated 12 adults according to the cluster of the study guidelines. In each family only one person was examined. If there were more than one qualified person, only one person was assessed randomly according to the checklist. In each cluster, 12 individuals in six age groups (15–25, 26–35, 36–45, 46–55, 56–65, 66 yr and above), and from each age group, one male and one female were evaluated. The questioning was lasted for 2 months (21). The data were analyzed by SPSS-20 software (Chicago, IL, USA).

## Results

Table 1 shows the prevalence of drug use according to sex and its use pattern. 4.6% of individuals used Opium and its derivatives, 0.4% used Cannabis, 0.4% used Amphetamine stimulants, 6.1% used Sedatives drugs and 2.6% used Alcoholic drinks on a regular basis. Within the last 12 months 3.9% of individuals used opium and its derivatives, 0.4% used Cannabis and 0.3% used Amphetamine stimulants, 5.8% used Sedatives drugs and 1.9% used Alcoholic drinks. Men were high drug users (except Sedatives Drugs) compared to women.

**Table 1:** Distribution of relative frequency of drug use in Iran based on sex and use pattern

***Type of drug/***	***Sex***		***Sample size***	***% of use throughout life***	***% of use within the last 12 months***	***Use pattern***		
						***1–3 times per months***	***1–3 times Per week***	***1–3 times per day***
Opium and its derivatives		Men	16887	7.5	6.3	3.23	1.54	3.06
		Women	16953	1.7	1.5	0.72	0.30	0.85
		Total	33830	4.6	3.9	1.99	0.93	1.97
Cannabis (Grass, Bang, Hashish)		Men	13796	0.7	0.6	0.63	0.19	0.26
		Women	13867	0.2	0.2	0.5	0.3	0.01
		Total	27663	0.4	0.4	0.34	0.11	0.14
Amphetamine stimulants (Methamphetamine, Ecstasy, Ritalin)		Men	16853	0.6	0.5	0.31	0.19	0.25
		Women	16944	0.2	0.1	0.04	0.04	0.03
		Total	33797	0.4	0.3	0.18	0.12	0.14
Sedatives Drugs (Diazepam, lorazepam, etc.)		Men	16856	5.5	5.2	1.57	1.55	3.09
		Women	16954	6.7	6.4	1.58	1.73	4.03
		Total	33810	6.1	5.8	1.58	1.64	3.56
Alcoholic drinks (Beer, sweat, wine)		Men	16821	4.6	3.3	2.62	0.49	0.21
		Women	16912	0.6	0.5	0.28	0.05	0.02
		Total	33733	2.6	1.9	1.46	0.27	0.12

Table 2 shows the province distribution of the drug use disorder prevalence in the last 12 months in the provinces of Iran. The highest prevalence of opium and its derivatives use was in the province of Kerman and the lowest was related to Qom Province. In relation to cannabis, the highest percentage of prevalence was belonging to Tehran province, and the least percentage of consumption was related to East Azerbaijan Province. The highest prevalence of Amphetamine Stimulants was in province of Sistan & Baluchistan, and the lowest was in the East Azerbaijan. The most prevalent use of sedative drugs was related to Kerman Province, and the lowest percentage was in Bushehr. Concerning consumption of alcoholic beverages, the highest prevalence rate was in Tehran Province and the lowest rate was related to Qom Province.

**Table 2:** Distribution the prevalence of all types of drugs used in the last 12 months in provinces of Iran

***Provinces***	***% use of Opium and its derivatives***	***% use of Cannabis***	***% use of Amphetamine Stimulants***	***% use of Sedatives Drugs***	***% use of Alcoholic Drinks***
West Azarbayjan	2.3	0.2	0.3	3.7	0.6
East Azarbayjan	2.7	0.3	0.1	3.3	1.4
Ardabil	2.0	0.2	0.1	4.2	0.6
Esfahan	2.9	0.3	0.1	4.5	1.7
Alborz	2.3	0.4	0.3	6.1	1.6
Ilam	2.3	0.3	0.3	5.6	3.7
Bushehr	3.0	0.4	0.3	3.0	1.2
Tehran	5.9	0.9	0.6	5.7	5.5
Chaharmahal & Bakhtiari	2.3	0.2	0.2	6.3	1.3
South Khorasan	4.4	0.6	0.3	6.1	0.9
Razavi Khorasan	3.3	0.5	0.3	6.2	1.4
North Khorasan	4.3	0.7	0.3	4.9	2.5
Khuzestan	3.8	0.7	0.3	4.6	1.4
Zanjan	3.5	0.3	0.2	5.7	0.9
Semnan	2.1	0.4	0.2	4.9	0.9
Sistan & Baluchistan	7.6	0.7	0.7	4.8	0.8
Fars	2.4	0.3	0.2	5.4	0.9
Ghazvin	6.0	0.2	0.7	7.6	3.2
Qom	1.2	0.2	0.1	5.6	0.1
Kordestan	1.9	0.4	0.2	5.2	3.6
Kerman	14.5	0.5	0.2	8.9	4.6
Kermanshah	4.5	0.2	0.2	6.2	1.1
Kohgilooye Buyer	2.8	0.4	0.4	5.4	1.7
Ahmad					
Golestan	4.4	0.3	0.3	4.6	1.7
Gilan	3.4	0.4	0.2	5.6	2.5
Lorestan	3.5	0.4	0.2	6.1	1.1
Mazandaran	2.0	0.5	0.3	5.0	1.2
Markazi	3.9	0.3	0.2	5.6	1.4
Hormozgan	3.4	0.4	0.2	6.2	1.6
Hamadan	7.8	0.3	0.9	7.7	3.5
Yazd	4.5	0.4	0.2	5.9	1.5
IRAN	3.9	0.4	0.3	5.8	1.9

## Discussion

The results of this study showed that 4.6% of individuals used opium and its derivatives throughout their life, and 3.9% of them used substances within the last 12 months. Although, the generalization of the findings of this study with other studies conducted in Iran is not logical due to the different statistical population, various tools used and different method of data collection, however comparing the findings of this research with studies presented in Table 3, demonstrated that the rate of substance abusers is fluctuating and increasing in Iran. Among the studied substances (opium, Cannabis and Amphetamine Stimulants), the prevalence rate of opium and its derivatives is higher, which is consistent with the results of other studies (10–15), however this result does not match with the report of the United Nations Office on Drugs and Crime, which indicates that the prevalence rate of Amphetamine stimulants is higher than other drugs (7). More people have access to opium and its derivatives because of the transit of these substances in Iran, might be one of the reasons for the high prevalence of opium and its derivatives in Iran.

**Table 3:** Comparison of prevalence rates of drug abuse researches in Iran

***Title***	***Year***	***Sample size Total***	***Statistical population***	***Prevalence rate %***	***Approximate number of addicted***
Epidemiology of drug Abuse in Iran	2001	5254	Patients aged 15 years and above from Emergency clinics in Iran	2.1	1158000
Rapid Assessment of Addiction in Iran	2004	4859	Clients of treatment centers, prisons and street addicts	2.4–2.8	1200000–1800000
Rapid Assessment of Addiction in Iran	2007	7731	Clients of treatment centers, prisons and street addicts	2.2–2.4	1100000–1200000
Epidemiology of drug abuse in Iran: National Survey of Mental Health	2010–2011	7886	Population aged 15–64 yrs.	2.8	1500000
The Epidemiology of drug use among citizens of Iran	2011	15000	Population aged 15–64 yrs.	2.65	1325000

Based on Table 1, the prevalence rate of opium and its derivatives within the last 12 months in men were 4.2 times than women, which are consistent with the findings of studies on the prevalence of addiction in Iran (11–13). Iincidence of drug use in women in recent years were higher than that of men, which was consistent with findings of other epidemiological studies of substance abuses in Iran. According to the results of table 1, with the exception of Sedatives drugs men used more drugs than women. Because the prevalence of mental disorders was more common in women than men in Iran, the rate of using sedatives drugs is more common in women than men due to their biopsychosocial problems within the society and comorbidity of mental disorders and social impairments (14, 17). 1.9% of the individuals used Alcoholic beverages within the last 12 months, which 0.39 % of them were alcoholic dependent. The results also indicated that within the last 12 months, the prevalence of opium and its derivatives were more common among rural inhabitants, the age group of 65 and over, divorced and widowed, under diploma, retired, and unemployed than other groups, which were consistent with the findings of Iranian Mental Health Survey on illicit drug use disorders (22). The higher prevalence rate in these groups could be due to the elderly problems, retirement, unemployment and weakness on usage of life skills to solve their problems.

Table 2 indicates that Kerman and Sistan & Baluchistan provinces were among the highest prevalence rate of Opium and its derivatives, which is consistent with the results of the study was done in 2011 (15). This higher rate might be due to being these provinces on the pathway of drug trafficking from two main drug producing countries, Afghanistan and Pakistan.

The weighted prevalence of any substance consumed in the population studied within the last 12 months, indicated that the number of addicts used opium and its derivatives were 2340000. The number of Cannabis, Amphetamine stimulants and Sedatives Drugs were 240000, 180000 and 3480000 individuals, respectively. Comparing the results (Table 3), demonstrated an increase of substance abuses in Iran, which requires health authorities should take all necessary measures to deal with these serious social problems.

### Recommendation

The method used in this cross-sectional study was a self-report study using ASSIST questionnaire, standardized for Iranian society (23). However, for standardization of ASSIST instrument, similar researches should be done by using other existing valid questionnaires to get cut-off point for Iranian society. For obtaining the trend of addiction within the society, this research should be repeated every two years. Implementing training workshops to increase knowledge, change attitude and improve performance of population in prevention of addiction also recommended.

## Conclusion

The rate of substance abusers is increasing in Iran. The increase of opium and its derivatives abusers in Iran, demands for actions by government in terms of policy formulation and addressing socioeconomic factors contributing to their use. Interventions directed to specific age groups, gender, and provinces are needed in order to reverse the current trend. Society needs to be educated to prevent their population from social and economic problems. Interventions that address the high level of drug availability and use are urgently needed. Modification of family monitoring may be effective in reducing drug use by elderly and adolescents, in addition to implementation of interventions that address eliminate unemployment and treatment of their illnesses, especially bio-psychological problems.

## Ethical considerations

Ethical issues (Including plagiarism, informed consent, misconduct, data fabrication and/or falsification, double publication and/or submission, redundancy, etc.) have been completely observed by the authors.
